# Regulation of Energy Homeostasis *via* GPR120

**DOI:** 10.3389/fendo.2014.00111

**Published:** 2014-07-11

**Authors:** Atsuhiko Ichimura, Takafumi Hara, Akira Hirasawa

**Affiliations:** ^1^Department of Molecular Medicine and Therapy, Tohoku University Graduate School of Medicine, Sendai, Japan; ^2^Department of Pharmacogenomics, Graduate School of Pharmaceutical Sciences, Kyoto University, Kyoto, Japan

**Keywords:** GPR120, FFAR4, FFAs, metabolic syndrome, diabetes mellitus

## Abstract

Free fatty acids (FFAs) are fundamental units of key nutrients. FFAs exert various biological functions, depending on the chain length and degree of desaturation. Recent studies have shown that several FFAs act as ligands of G-protein-coupled receptors (GPCRs), activate intracellular signaling and exert physiological functions *via* these GPCRs. GPR120 (also known as free fatty acid receptor 4) is activated by unsaturated medium- to long-chain FFAs and has a critical role in various physiological homeostasis mechanisms such as incretin hormone secretion, food preference, anti-inflammation, and adipogenesis. Recent studies showed that a lipid sensor GPR120 has a key role in sensing dietary fat in white adipose tissue and regulates the whole body energy homeostasis in both humans and rodents. Genetic study in human identified the loss-of-functional mutation of GPR120 associated with obesity and insulin resistance. In addition, dysfunction of GPR120 has been linked as a novel risk factor for diet-induced obesity. This review aims to provide evidence from the recent development in physiological function of GPR120 and discusses its functional roles in the regulation of energy homeostasis and its potential as drug targets.

## Introduction

Free fatty acids (FFAs) are basic components of biological structures, precursors of various mediators, and play important roles as essential nutrients (1). During the past decade, however, a number of studies revealed that FFAs also act as key signaling molecules to regulate a number of physiological functions through G-protein-coupled receptors (GPCRs) (1–4). The superfamily of GPCRs includes at least 800 seven-transmembrane receptors that have diverse physiological and pathological functions. GPCRs are the most successful targets of drug (5). Of interest, FFAs act as ligands of some GPCRs. FFAs can be classified depending on their chain length as short-chain fatty acids (SCFAs), which have 1–6 carbon chain length; medium-chain fatty acids (MCFAs, 7–12 carbon chain length); and long-chain fatty acids (LCFAs), which have more than 12 carbon chain length. Some of non-esterified FFAs directly regulate important biological processes such as energy homeostasis *via* their corresponding receptors (6–10). The LCFA receptor GPR40 (also known as FFAR1), SCFA receptors GPR41 (FFAR3) and GPR43 (FFAR2) were identified in 2003 (11–18). In 2005, we successfully deorphanized and identified GPR120 [also known as free fatty acid receptor 4 (FFAR4)] as a FFAs receptor (FFARs), which is activated by unsaturated MCFAs and LCFAs (19). These GPCRs are widely expressed in the body and contribute to maintain systemic energy homeostasis under changing nutritional conditions. Among these FFARs, GPR120 emerged as an important checkpoint in regulating energy homeostasis (6, 8). Previous studies also showed that GPR120 has been implicated in several key processes including the release of incretin hormone, anti-inflammation, food preference, glucose homeostasis, insulin sensitivity, and adipogenesis (6, 8, 19–24). These factors interrelate to regulate systemic metabolic energy and nutritional homeostasis under physiological and pathophysiological conditions. Hence, in this review, we attempt to summarize and discuss the recent advances in research regarding the roles of GPR120.

## Tissue Distribution of GPR120

GPR120 is widely expressed in various tissues and cell types including intestine, macrophages, adipose tissue, taste buds, brain, pancreas, lung, thymus, and pituitary (2, 6, 8). Hence, GPR120 has multiple functions in homeostatic regulation of systemic metabolism and inflammation depending on this diverse tissue distribution. Furthermore, GPR120 is co-localized with not only glucagon-like peptide 1 (GLP-1) in the colon and circumvallate papillae taste bud cells (19, 25, 26), but also with ghrelin (27) and α-gustducin in the duodenum and type II taste bud cells, respectively (28, 29). GPR120 was also reported to be co-expressed with other FFARs, such as GPR40 in STC-1 intestinal cells (19) and GPR43 in the proximal colon in mice (29). These characteristics of expression patterns and co-localization might reflect the physiological functions of GPR120 as described below.

## Intestine

GPR120 is expressed in the intestines of humans as well as mice. Furthermore, the enteroendocrine cell line STC-1 also expressed GPR120 endogenously. We have previously shown that GLP-1-expressing enteroendocrine cells in the colon were expressing GPR120 in both rodents and human (19, 20, 25). Secretion of GLP-1 and cholecystokinin (CCK), both known as incretin hormones and involved in the regulation of feeding behaviors, energy metabolism and bodyweight (30–32), was induced by FFAs stimulation from enteroendocrine STC-1 cells (33). The administration of FFAs into the murine colon stimulated GLP-1 secretion and increased plasma level of insulin (19). Furthermore, we have found that the knockdown of GPR120 expression by siRNA inhibited the FFAs-induced [Ca^2+^]_i_ response and incretin hormones secretion in STC-1 cells. These data highly suggested that GPR120 indeed mediate and stimulate incretin hormone secretion *in vivo*. In addition, K cells, which are found in the mucosa of the duodenum and the jejunum of the gastrointestinal tract and also synthesize gastric inhibitory peptide (GIP), also express GPR120 (34). Interestingly, recent reports indicated that GPR120 was co-localized with the orexigenic peptide, ghrelin in duodenal cells *in vivo*, and FFAs stimulation reduced ghrelin secretion in the MGN31 ghrelinoma cell line (35). Furthermore, Gong *et al*. revealed that addition of GW-9508, a GPR120 chemical agonist, inhibited the secretion of ghrelin from ghrelin-producing stomach ghrelinoma (SG-1) cells. They also showed that SG-1 cells highly expressed GPR120 endogenously and the inhibitory effect of GW-9508 on ghrelin secretion was blocked by siRNA against GPR120 in SG-1 cells. Furthermore, GW-9508 treatment reduced plasma ghrelin level *in vivo* (36). These reports indicate that the decrease of postprandial ghrelin is induced at least partially by LCFAs included in foods *via* GPR120. Given the effects on GLP-1, CCK, and ghrelin secretion, the stimulation of GPR120 might regulate appetite and systemic energy homeostasis.

## Macrophages

GPR120 was found to be expressed in monocytic RAW267.4 cells and primary proinflammatory M1-like macrophages (6). The activation of GPR120 by ω-3 LCFAs, such as docosahexaenoic acid (DHA) and alpha-linolenic acid (α-LA), exerts broad of anti-inflammatory effects in these cells, all of which were abolished by siRNA against GPR120. These ω-3 LCFAs are identified as anti-inflammatory fatty acids in the tissue-specific and systemic levels (9). Oh *et al*. clearly showed that ω-3 LCFAs exert anti-inflammatory effects through GPR120. *In vitro* experiments revealed the molecular mechanism underlying ω-3 FFAs-mediated anti-inflammatory effects. Stimulation of GPR120 by ω-3 LCFAs abolished lipopolysaccharide (LPS)-induced phosphorylation and activation of IκB kinase (IKK) and c-Jun N-terminal kinase (JNK) in macrophages. Recruitment of β-arrestin 2 (β-arr2) and following the GPR120–β-arr2 complex internalization is induced by the activation of GPR120. Tumor necrosis factor-α (TNF-α) and toll-like receptor 4 (TLR4) widely mediate proinflammatory cascades. In addition, tumor growth factor β (TGF-β) activated kinase 1 (TAK1) interacting with TGF-β activated kinase 1 binding protein 1 (TAB1) mediate downstream inflammatory effects *via* activation of NF-κB and JNK. The internalized GPR120–β-arr2 complex interacts with TAB1 and inhibits the interaction between TAB1 and TAK1, leading to the inhibition of the downstream proinflammatory pathways. Further *in vivo* experiments demonstrated that administration of ω-3 FFAs ameliorated tissue inflammation and thereby improved systemic insulin sensitivity in wild type (WT) mice. The gene deficiency of GPR120 abolished these effects of ω-3 FFAs (6, 9, 37). These results showed that the activation of GPR120 by ω-3 FFAs exerts potent insulin sensitizing and anti-diabetic effects *in vivo* by the repression of macrophage-induced tissue inflammation.

## Adipose Tissue

GPR120 was also found to be expressing endogenously in adipocyte and adipose tissue, but not detected in pre-adipocyte (8, 22). Furthermore, GPR120 expression was increased according to the lipid accumulation in the cells during induction of adipocyte differentiation in 3T3-L1 cells (22). Knockdown and gene deficiency of GPR120 by siRNA suppressed the expression of adipogenic genes and lipid accumulation in 3T3-L1 cells and mouse embryonic fibroblast, respectively (8, 22). These data indicated that GPR120 might be an adipogenic receptor and might play important roles in adipocyte differentiation and maturation. GPR120 mRNA expression was increased in subcutaneous, epididymal, and mesenteric adipose tissue of high fat diet (HFD)-fed mice (22). Moreover, we have shown that GPR120 expression in human adipose tissue was significantly higher in obese individuals than in lean controls (8), suggesting that the expression of GPR120 could be enhanced by the accumulation of dietary lipid in both rodent and human. Our previous study revealed that GPR120-deficient mice fed HFD developed obesity, which was accompanied with decreased differentiation and lipogenesis in adipocyte. Furthermore, severe fatty liver, enhanced hepatic lipogenesis, increased fasting glucose, and impaired responses to insulin and glucose tolerance were observed in HFD-fed GPR120-deficient mice. Gene expression analysis in adipose tissue and liver revealed the molecular basis underlying obesity and insulin resistance of GPR120-deficient mice. Our data showed that HFD-fed GPR120-deficient mice showed a significantly decreased expression of adipogenic gene Fabp4 as well as the key lipogenic gene Scd1. In addition, macrophage marker genes were also increased in adipose tissue, an indication of adipose tissue inflammation. In the liver, on the other hand, the key lipogenic gene Scd1 expression was significantly increased. Insulin signaling-related genes were significantly decreased in both adipose tissue and the liver of HFD-fed GPR120-deficient mice. Furthermore, phosphorylation of IRβ and IRS1 in white adipose tissues and IRS1 and IRS2 in the liver, all of which are regulators of insulin-stimulated glucose uptake, were significantly decreased. In addition, Oh *et al*. reported that GPR120 induced a translocation of glucose transporter 4 in 3T3-L1 adipocytes and directly increased glucose uptake (6). Taking together, these data demonstrated that GPR120 acts as a lipid sensor *in vivo* and plays a critical role in sensing dietary fat to regulate glucose and lipid metabolism.

## Taste Buds

Recent studies strongly suggested that oral perception of dietary fat was involved in the detection of taste, in addition to texture and olfaction, of LCFAs (38). GPR120 was reported to be expressed in taste bud type II cells (28). Matsumura *et al*. showed the co-localization of GPR120 with phospholipase-Cβ2 and α-gustducin in the taste buds by double immunostaining. Cartoni *et al*. further showed the expression of GPR120 in circumvallate papillae (CV) sections by immunohistochemical analysis (39). Short-access test using a lick meter showed that gene deficiency of GPR120 abolished the preference for fatty acids but not for other tastes. These data suggest that the upregulation of GPR120 in the taste buds could induce an excess intake of lipid, leading to obesity. Martin *et al*. also reported that GPR120 and GLP-1 were found to be co-localized in mouse taste cells from mouse CV (26). Studies using GPR120 selective agonist and isolated mouse CV indicated that GPR120 might be responsible for LCFAs-mediated release of GLP-1 from CVs and might thus contribute to the high palatability of foods rich in both fats and sugars. A recent study further showed that human primary taste bud cells were expressing GPR120 (40). High concentrations of linoleic acid induced [Ca^2+^]_i_ signaling *via* GPR120 and CD36 in human and mice primary taste bud cells. These reports strongly suggested that GPR120 expressed in taste buds plays an important role in sensing fat taste, contributing to the food intake.

## Other Tissues

GPR120 is also expressed in other tissues and cells. Cintra *et al*. performed immunostaining analysis and found that GPR120 co-localized with neuropeptide Y centrally in the arcuate nucleus (41). An acute injection of ω-3 and ω-9 FFAs-induced GPR120–β-arr2 complex and β-arr2–TAB1 complex as well as inhibited the interaction between TAB1 and TAK1, leading to the reduction of the downstream proinflammatory pathways in the hypothalamus. Furthermore, Wellhauser *et al*. analyzed the molecular mechanisms to modulate hypothalamic function *via* GPR120 *in vitro* using a hypothalamic neuronal model, rHypoE-7 cells, isolated from the rat. They showed that the anti-inflammatory effect of DHA was significantly reduced by siRNA against GPR120 in rHypoE-7 cells (42). Numbers of studies showed inflammatory response in the hypothalamus in reaction to excessive nutrients contributes to diet-induced obesity and type 2 diabetes mellitus (43–45). Hence, the anti-inflammatory effect mediated by GPR120 in hypothalamus might play an important role in the regulation of systemic energy homeostasis.

Recently, Xhao *et al*. showed mRNA and protein expression of GPR120 in human and rat pancreas (46). Immunohistological analysis demonstrated that GPR120 is co-localized with CD68, the specific marker of macrophages, and with CD34 and CD117, the markers of interstitial cells in the pancreas. Furthermore, Stone *et al*. generated Gpr120-knockout/β-galactosidase knock-in mice and showed the distribution of GPR120 (23). Immunofluorescence analysis demonstrated the co-localization of GPR120 with somatostatin, suggesting that GPR120 is selectively expressed in islet delta cells. They also demonstrated that treatment of GPR120 selective antagonist inhibited glucose induced somatostatin secretion from isolated islet. Additionally, GPR120-deficiency abolished this effect. Hence, GPR120 expressed in pancreatic delta cells might regulate somatostatin secretion. Further studies are required in order to reveal the functional roles of GPR120 in pancreas.

## Genetic Contribution to Type 2 Diabetes

We previously reported two non-synonymous mutation p.R270H and p.R67C by exon sequencing of GPR120 in obese and lean European subjects. Following *in vitro* experiments revealed that the p.R270H mutant, which significantly associated with obesity, lacked the ability to transduce LCFAs signal, contrary to the p.R67C mutant, which was not associate with obesity. Taken together these human and GPR120-deficient mice, the dysfunction of GPR120 leads to obesity in both mice and human (8). In addition, the systems genomics approach to identify genes for type 2 diabetes showed that GPR120 was placed in the top 16 of the ranked list (47). Taneera *et al*. reported that GPR120 expression in human islets was positively correlated with both secretion and contents of insulin as well as lower HbA1c levels. These data suggested that GPR120 might have a protective role on human islet.

## Conclusion

**Figure 1:**
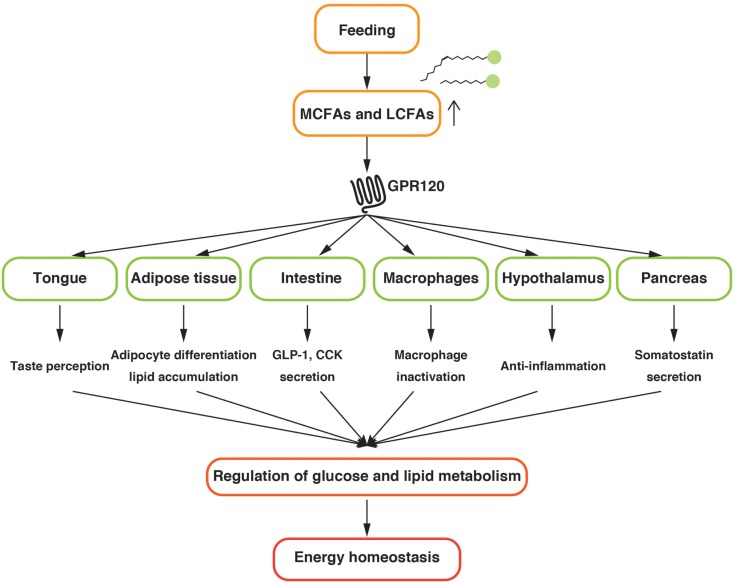
**Schematic diagram of the physiological function of GPR120 related to the energy homeostasis**.

GPR120 regulates the metabolic homeostasis by sensing LCFAs provided by dietary fat in several tissues (Figure 1). Further investigations to uncover the precise physiological functions of GPR120 are mandatory for a better understanding of systemic nutrient metabolism and energy homeostasis. The current studies suggest that GPR120 activation might have positive outcomes on health. Hence, GPR120 might be a promising pharmaceutical target for the treatment of metabolic diseases.

## Conflict of Interest Statement

The authors declare that the research was conducted in the absence of any commercial or financial relationships that could be construed as a potential conflict of interest.
